# Tumor-infiltrating CD62L^+^PD-1^-^CD8 T cells retain proliferative potential via Bcl6 expression and replenish effector T cells within the tumor

**DOI:** 10.1371/journal.pone.0237646

**Published:** 2020-08-26

**Authors:** Yu Gong, Toshihiro Suzuki, Haruo Kozono, Masato Kubo, Naoko Nakano

**Affiliations:** 1 Research Institute for Biomedical Sciences, Tokyo University of Science, Chiba, Japan; 2 School of Medicine and Faculty of Medical Technology, General Medical Education and Research Center, Teikyo University, Tokyo, Japan; Maisonneuve-Rosemont Hospital, CANADA

## Abstract

Tumor antigen–primed CD8 T cells differentiate into effector T cells that kill tumor cells rapidly, whereas durable responses of CD8 T cells are required to cope with long-lasting tumor growth. However, it is not well known how persisting CD8 T cells are generated. In this study, we analyzed CD8 T cells primed by antigens in tumor-draining lymph nodes and found that CD8 T cells first differentiated into a CD62L-intermediate (CD62L^int^) stage upon antigen stimulation. These cells gave rise to tumor-infiltrating CD62L^-^CD44^high^ Bcl6^-^ effector T cells and CD62L^+^CD44^high^Bcl6^+^ memory-like T cells. Memory-like T cells within the tumor expressed CD127, CXCR3 and had the potential to proliferate significantly when they were transferred into tumor-bearing mice. Bcl6 expression in these T cells was critical because Bcl6^-/-^CD62L^+^CD44^high^CD8T cells within the tumor were defective in expansion after secondary transfer. Taken together, our findings show that CD62L^+^CD44^high^Bcl6^+^ cells are generated from highly proliferating CD62L^int^ T cells and retain high proliferative potential, which contributes to replenishment of effector T cells within the tumor.

## Introduction

Antigen priming of CD8 T cells is crucial to induce effector T cells that eliminate viral infections and tumor cells. Following contraction of the effector T cell population, a limited number of T cells are maintained as memory T cells. Memory T cells can be categorized as CD62L^+^ central memory T cells, which have self-renewal potential, CD62L^-^ effector memory T cells, and non-circulating tissue-resident memory T cells [1]. Antigen-primed CD8 T cells exhibit transcriptional divergence in the progeny of their first division [2]. Factors that drive the effector versus memory CD8 T cells could be signal strength through TCR stimulation [3] and cytokines such as IL-2 [4] and IL-15 [5].

In anti-tumor CD8 T cell responses, as well as in chronic viral infection, persistent antigens promote altered T cell differentiation, resulting in generation of effector T cells with memory phenotypes with stem-like properties [6–9]. These stem-like CD8 T cells, which express Tcf1 and sustain immune responses, have the potential to expand in response to PD-1 blockade. However, it is not well understood how these T cells are generated during antigen priming.

B cell lymphoma 6 protein (Bcl6) was identified as a differentiation factor for follicular helper T cells [10–12], and Bcl6 expressed in CD8 T cells is required for the generation and maintenance of memory T cells [13]. Bcl6 promotes the expression of Tcf1 in acute viral infection [14]. Bcl6 represses genes encoding molecules involved in the glycolysis pathway, which is required for effector T cell differentiation [15], thereby supporting the memory T cell differentiation pathway.

In this study, we analyzed tumor-infiltrating CD8 T cells that express intermediate levels of CD62L. These CD62L^+^ T cells were Bcl6^+^ and generated directly from CD62L^int^CD44^high^ Bcl6^+^ T cells in tumor-draining lymph nodes. Tumor-infiltrating CD62L^+^ Bcl6^+^ T cells did not express PD-1, and had a high potential to expand and differentiate into effector T cells. Lack of Bcl6 in tumor-infiltrating CD62L^+^ T cells impaired the ability to expand. Consequently, CD62L^int^CD44^high^ T cells that appeared upon antigen priming in tumor-draining lymph nodes maintained their potential for expansion by expressing Bcl6 after tumor infiltration. Targeting these CD62L^int^CD44^high^ T cells in addition to the checkpoint blockade represents a new strategy for inducing tumor immunity.

## Materials and methods

### Mice

C57BL/6J mice were purchased from Sankyo Laboratories (Shizuoka, Japan). *Bcl6*-floxed mice [16] and *Cd4-Cre* mice [17] were described previously. OT-1 TCR transgenic mice (CD45.2) were crossed with C57BL/6J (CD45.1) to generate OT-1(CD45.2/CD45.1) mice. Seven- to ten-week-old male or female mice were used in the experiments. All mice were maintained under specific pathogen–free conditions in the animal facility at Tokyo University of Science, and experimental studies were approved by the university’s Animal Care and Use Committee. (Permit No. S19028)

### Antibodies and reagents

Antibodies against CD62L (MEL-14), CD127 (A7R34), KLRG1 (2F1), PD-1 (9F.1A12), CD69 (H1.2F3), CD44 (M7), B220 (RA3-6B2), CD11c (N418), CD11b (M1/70) CD4 (GK1.5), CD8 (53–6.7), CXCR3 (CXCR3-173), CD45.1 (A20), CD45.2 (104), and Bcl6 (7D1), conjugated to FITC, PE, PE-Cy7, APC, Pacific blue, or biotin, were purchased from BioLegend. Alexa Fluor 647-conjugated antibody against TCF1 (clone#812145) and isotype control (clone#141945) were purchased from R&D systems. CD16/32 (2.4G2) antibodies were used for Fc blocking. To generate OVA-H-2K^b^ tetramer, H-2K^b^ molecules connected with the FLAG-tag and the BirA recognition sequence were expressed in Sf9 cells using the Bac-to-Bac system (Invitrogen). Molecules purified with M2 affinity gels (Sigma) were mixed with OVA_257-264_ peptides (SIINFEKL) to form the MHC/peptide complex and biotinylated with BirA enzyme, which were then bound to Alexa Fluor 647–labeled streptavidin. Carboxyfluorescein succinimidyl ester (CFSE) was purchased from DOJIN, Japan.

### Tumor transplantation

Lewis lung carcinoma cells and B16 melanoma cells transduced with the ovalbumin gene (LLC-OVA and B16-OVA) were generated as described previously [18]. Cells (1×10^6^) were injected intradermally into the flanks of mice.

### Tumor volume measurement

In the experiments testing the ability to control tumor growth, 1×10^5^ B16-OVA were transplanted subcutaneously. Two days later, antigen primed OT-1 T cells were transferred and tumor sizes were measured every two days. Tumor volumes were measured using vernier calipers on the indicated days. All the measurements were performed three times, and an average of the three measurements was obtained. The tumor volume was calculated according to the formula (0.52 × length × width^2^).

### Adoptive T cell transfer

Naïve OT-1 T cells were obtained from spleen and lymph nodes of OT-1 mice. Cells prepared after hemolysis were incubated with biotin-labeled antibodies (anti-CD4, CD44, B220, CD11c, CD11b, TCRγδ), and biotin-labeled cells were eliminated with Streptavidin Particle Plus-DM (BD IMag, BD Biosciences). These T cells were intravenously transferred into 7-9-week-old sex-matched tumor-transplanted mice. Transferred OT-1 T cells in the tumor and tumor-draining lymph nodes were analyzed 1–3 weeks after transfer. In some experiments, OT-1 T cell populations within the tumor and tumor-draining lymph nodes were sorted, and these T cells were further transferred into tumor-transplanted mice.

### Flow cytometry and cell analysis

Tumor-infiltrating lymphocytes were obtained by incubating excised tumor tissues for 30 min at 37°C with 1 μg/ml Liberase TL, research grade (Roche) and 50 μg/ml DNase I, type II (Sigma). Cells were subjected to flow cytometry on a BD FACSCanto II or BD FACSCalibur II, and the resultant data were analyzed with the FlowJo software.

### Statistics

Statistical analyses were performed using Student’s *t* test, two-tailed, unpaired, using GraphPad prism version 7.0. A *p* value < 0.05 was considered statistically significant.

## Results

### Tumor-infiltrating CD8 T cells expressing Bcl6 persist within the tumor

**Fig 1:**
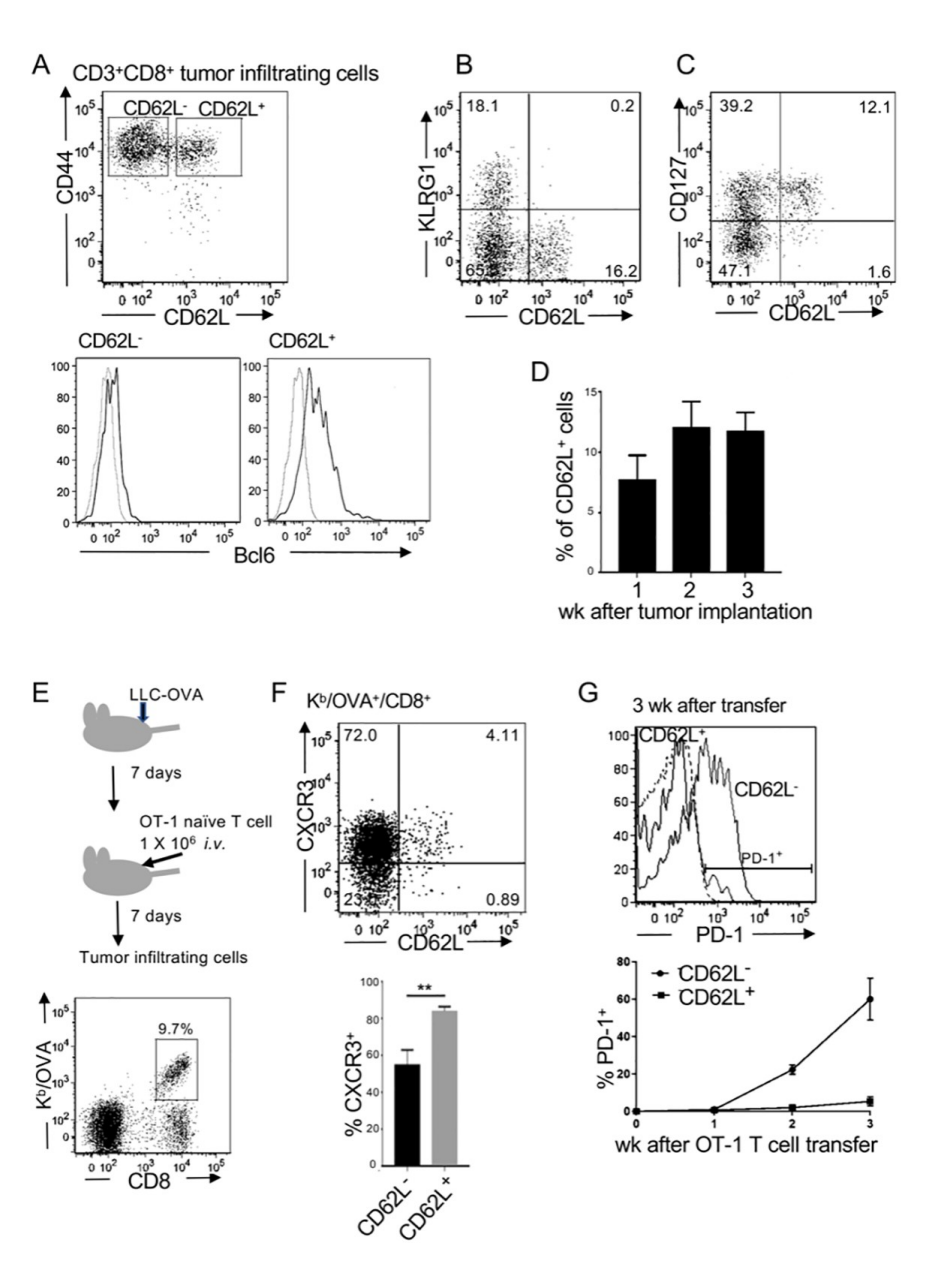
Memory phenotype CD8 T cells expressing Bcl6 persist within the tumor. (A–C) C57BL/6 mice were intradermally transplanted with LLC-OVA. Representative flow cytometry plots and histograms of tumor-infiltrating CD3^+^CD8^+^T cells on day 7 of three independent experiments are shown. (A) Bcl6 expression in sorted CD62L^+^ and CD62L^-^ cells. (D) Frequencies of CD62L^+^ cells within the CD3^+^CD8^+^ tumor-infiltrating cells. Representative data of three independent experiments are shown. (Mean ± SEM, n = 3) (E) Experimental design. OT-1 naïve T cells were adoptively transferred into C57BL/6 mice that had been transplanted with LLC-OVA 7 days previously. K^b^/OVA tetramer^+^ cells were analyzed on day 7. (F) Flow cytometric analysis of K^b^/OVA^+^ cells and frequencies of CXCR3^+^ cells. Representative data of three independent experiments are shown. (Mean ± SEM, *P* ** <0.01, n = 3) (G) PD-1 levels of CD62L^+^ and CD62L^-^ tumor-infiltrating OT-1 T cells 3 weeks after the transfer. A representative analysis of three independent experiments is shown (top). Frequencies of PD-1^+^ cells in CD62L^+^ and CD62L^-^ populations 1–3 weeks after OT-1 T cell transfer (bottom). Data are shown as mean ± SEM (n = 3). Representative data of three independent experiments.

Tumor-infiltrating CD8 T cells are heterogeneous due to various signals received from antigen-presenting cells and tumor microenvironment [19, 20]. We first analyzed tumor-infiltrating CD8 T cells and found that CD44^high^ cells could be divided into CD62L^+^ and CD62L^-^ populations. CD62L^+^CD44^high^ CD8 T cells expressed Bcl6 and had a central memory-like phenotype, expressing CD127 but not KLRG1 (Fig 1A, 1B and 1C). Bcl6^+^ cells were part of Tcf1^+^ cells in tumor-infiltrating CD8 T cells (S1 Fig). Between 5% and 15% of tumor-infiltrating CD8 T cells were CD62L^+^ 1–3 weeks after tumor transplantation (Fig 1D). To determine whether these T cells were generated in an antigen-specific manner, native OT-1 T cells were transferred into C57BL/6 mice that had been intradermally transplanted with LLC-OVA 1 week previously. Tumor-infiltrating cells contained 5–10% OVA-specific CD8 T cells, which were stained with OVA-H-2K^b^ tetramer (K^b^/OVA) (Fig 1E). These CD62L^+^ cells preferentially expressed CXCR3 (Fig 1F), which allowed them to infiltrate into tumor tissues. Importantly, these CD62L^+^ OT-1 T cells remained PD-1 at low levels, whereas a large proportion of CD62L^-^ OT-1 T cells became PD-1^+^ at 3 weeks (Fig 1G). Expression of CXCR3 and lack of PD-1 in CD62L^+^ cells were commonly observed in tumor infiltrating polyclonal T cells (S2A and S2B Fig). These results suggested that CD62L^+^ T cells were not exhausted within the tumor.

### Antigen-primed CD8 T cells with intermediate levels of CD62L give rise to tumor-infiltrating CD62L^-^ effector and CD62L^+^Bcl6^+^ T cells

**Fig 2:**
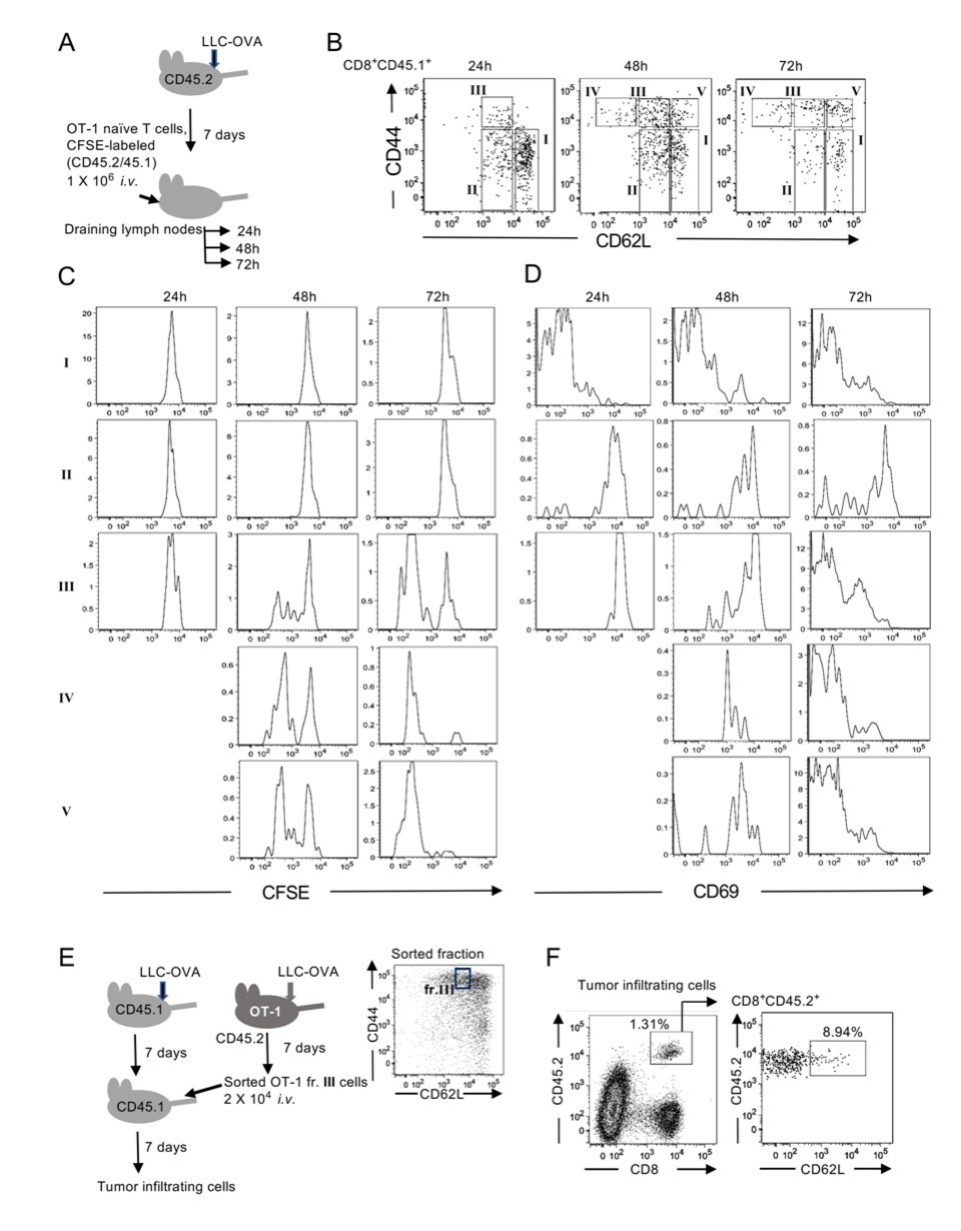
Antigen-primed CD62L^int^CD44^high^ T cells in tumor-draining lymph nodes give rise to CD62L^+^ and CD62L^-^ tumor-infiltrating T cells. (A) Experimental design. CSFE-labeled OT-1 naïve T cells (CD45.2/CD45.1) were adoptively transferred into C57BL/6 (CD45.2) mice that had been transplanted with LLC-OVA 7 days previously. (B) Representative flow cytometry plots of three independent experiments. CD8^+^CD45.1^+^ tumor-draining lymph node cells were divided into five populations (I–V) based on the expression levels of CD62L and CD44. (C) Cell division of transferred OT-1 T cells in the fractions shown in (B) was measured by CFSE dilution. (D) CD69 expression in cells in the fractions was analyzed. (E) Experimental design. Draining lymph node cells in CD62L^int^CD44^high^ (fr.III) from LLC-OVA transplanted OT-1 mice (CD45.2) on day 7 were sorted. Cells were adoptively transferred into wild-type mice (CD45.1) that had been transplanted with LLC-OVA 7 days previously. Tumor-infiltrating cells were analyzed on day 7. (F) Representative flow cytometry plots of tumor-infiltrating cells of three independent experiments are shown.

To determine how CD62L^-^ effector-type and CD62L^+^Bcl6^+^ memory-like T cells are generated, we analyzed activation and differentiation of antigen-primed OT-1 T cells in tumor-draining lymph nodes. Naive OT-1 T cells (CD62L^high^CD44^low^) purified from OT-1 in CD45.1/CD45.2 background mice were labeled with CFSE and transferred into LLC-OVA–transplanted CD45.2 mice (Fig 2A). OT-1 T cell responses in tumor-draining lymph nodes, gated with CD8 and CD45.1 expression, were analyzed 24, 48, and 72 h after OT-1 T cell transfer (Fig 2B). At 24 h, OT-1 T cells in tumor-draining lymph nodes were found in three fractions (I: CD62L^high^CD44^low^, II: CD62L^int^CD44^low^, III: CD62L^int^CD44^high^), and cells in all three fractions maintained CFSE at high levels (Fig 2C). Another two fractions, CD62L^low^CD44^high^ (IV) and CD62L^high^CD44^high^ (V), appeared 48 h after the transfer. A larger proportion of cells in these two fractions had divided two or three times already. Elevated numbers of OT-1 T cells in fraction III contained both divided and non-divided cells, and continuous division was observed in fractions III, IV, and V at 72 h. CD69 expression in T cells is induced upon activation, which promotes the internalization of S1P1 receptor, leading to the inhibition of egress of activated T cells [21]. Twenty-four hours after OT-1 T cell transfer, OT-1 T cells in fractions II and III expressed CD69, whose levels started to decline at 48 h in fraction III (Fig 2D). The newly appeared fractions IV and V expressed lower levels of CD69 than fraction III, suggesting that cells in fractions IV and V were more differentiated. At 72 h, CD69 expression in fractions III, IV, and V went down, and these cells became ready to leave the lymph nodes. In fact, all three fractions of cells were detected in the spleen at 72 h (S3 Fig), suggesting that antigen-primed T cells with different levels of CD62L had started to circulate within the body. Because fraction III, which had intermediate levels of CD62L, appeared first and contained fewer divided cells even after 72 h, we speculated that the cells in fractions IV and V were derived from those in fraction III. We then asked whether CD62L^int^CD44^high^ (III) cells could give rise to both CD62L^+^ and CD62L^-^ populations within the tumor. To test this idea, we sorted CD62L^int^CD44^high^ (III) cells in draining lymph nodes from OT-1 mice that had been transplanted with LLC-OVA 1 week previously (Fig 2E). These cells were still cycling, as reflected by expression of Ki67 (S4 Fig). The sorted CD62L^int^CD44^high^ (III) cells were transferred into tumor-transplanted wild-type (CD45.1) mice (Fig 2E). Tumor-infiltrating OT-1 T cells were detected by staining with anti-CD8 and CD45.2. The transferred OT-1 T cells were identified as CD62L^+^ and CD62L^-^ cells within the tumor (Fig 2F), indicating that CD62L^int^CD44^high^ cells differentiated into both effector and memory-like T cells.

### CD62L^+^BCL6^+^ T cells have a high potential to proliferate

**Fig 3:**
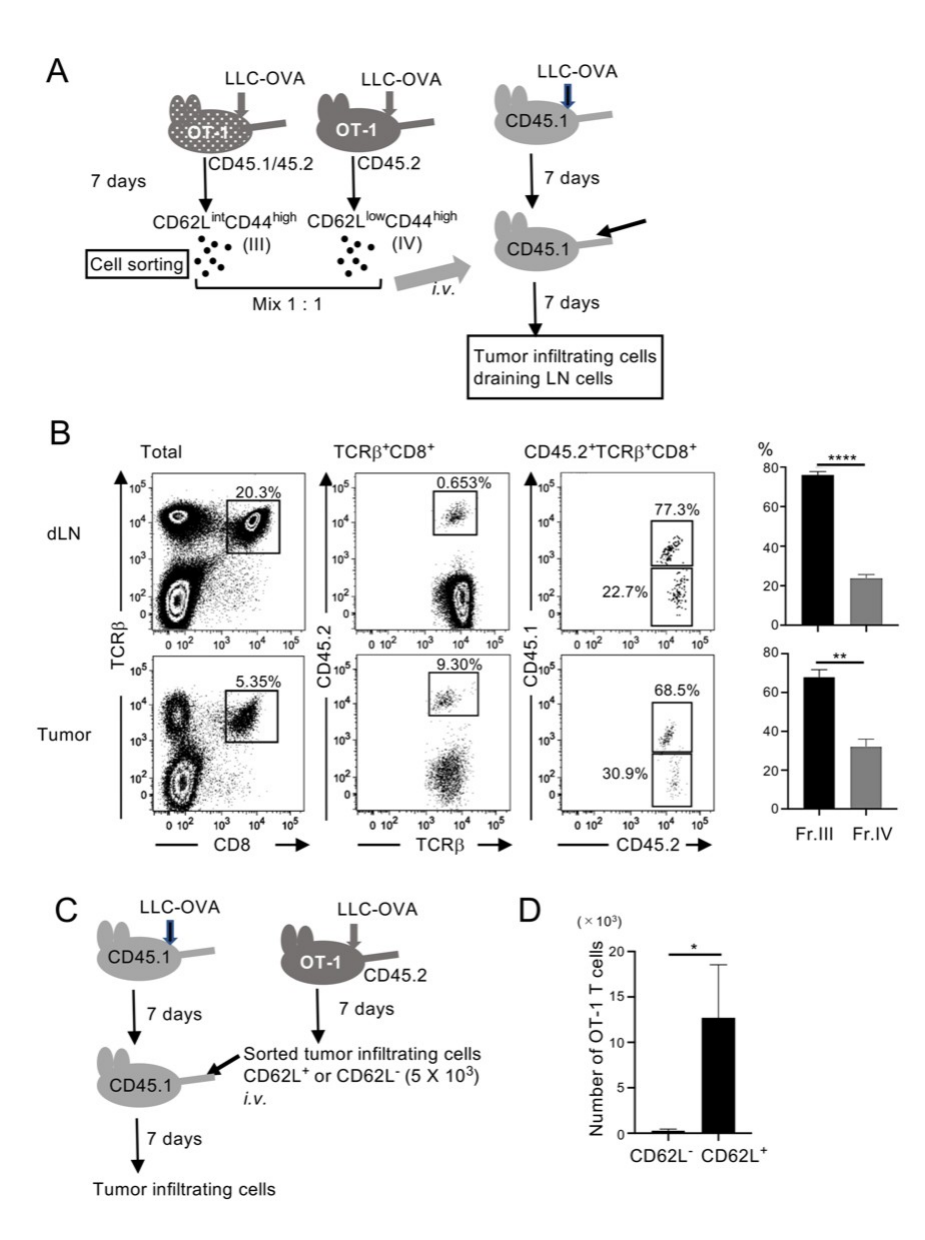
CD62L^int^CD44^high^ T cells both in tumor-draining lymph nodes and within the tumor have a strong potential to proliferate. (A) Experimental design. K^b^/OVA^+^CD62L^int^CD44^high^ (III) and CD62L^-^CD44^high^ (IV) cells in tumor-draining lymph nodes in LLC-OVA–transplanted OT-1(CD45.1/45.2) and OT-1(CD45.2), respectively, were sorted. Cells were mixed at a 1:1 ratio and transferred *i*.*v*. into LLC-OVA–transplanted wild-type (CD45.1) mice. Transferred OT-1 T cells in the tumor and tumor-draining lymph nodes were analyzed on day 7. (B) Representative flow cytometry plots of two independent experiments. Frequencies of CD45.1^+^ cells (from fr.III) and CD45.1^-^ cells (from fr.IV) among TCRβ^+^CD8^+^ CD45.2^+^ cells were analyzed. Mean ± SEM; **** *P*<0.001, ** *P*<0.01; n = 3. (C) Experimental design. K^b^/OVA^+^CD62L^+^ and CD62L^-^ tumor-infiltrating cells were sorted from LLC-OVA–transplanted OVA mice. Cells (5 × 10^3^) were transferred into LLC-OVA–transplanted wild-type (CD45.1) mice. Tumor-infiltrating cells were analyzed on day 7. (D) Numbers of cells derived from transferred OT-1 T cells within the tumor were calculated from the cell numbers passed through the FACS and their profiles. Data shown are representative of two independent experiments. Mean ± SEM; * *P*<0.05; n = 3.

Although antigen-primed CD62L^int^CD44^high^ (III) cells gave rise to tumor infiltrating CD62L^+^ and CD62L^-^ cells, CD62L^low^ CD44^high^ (IV) cells in tumor-draining lymph nodes could be a major source of highly activated CD8 T cells, which kill tumor cells. To determine which fraction of cells in tumor-draining lymph nodes makes the greatest contribution, we sorted cells in fractions III and IV from tumor-draining lymph nodes in LLC-OVA–transplanted OT-1 (CD45.1/CD45.2) and OT-1 (CD45.2) mice, respectively. Sorted cells were mixed at 1:1 ratio and transferred *i*.*v*. into LLC-OVA transplanted wild-type (CD45.1) mice. Cells in tumor-draining lymph nodes and tumors were analyzed 7 days after the transfer (Fig 3A). Among the transferred T cells detected as CD45.2^+^, cells originating from fraction III that expressed CD45.1, were 2–3 times more abundant than those originating from fraction IV (CD45.1^-^), within both the tumor and lymph nodes (Fig 3B). These results indicated that antigen-primed CD8 T cells that express intermediate levels of CD62L had a greater potential to proliferate and gave rise to both CD62L^+^ and CD62L^-^ T cells. To determine whether CD62L^+^ T cells within the tumor also have a high proliferation potential, we sorted CD62L^+^ and CD62L^-^ tumor-infiltrating K^b^/OVA^+^CD8^+^ T cells from LLC-OVA–transplanted OT-1 mice. These cells were transferred into LLC-OVA–transplanted wild-type (CD45.1) mice, and tumor-infiltrating OT-1 T cells were analyzed on day 7 (Fig 3C). In mice that received 5 × 10^3^ tumor-infiltrating CD62L^+^ OT-1 T cells, more than 1 × 10^4^ cells were found within the tumor, whereas few OT-1 T cells were detected in the mice that received 5 × 10^3^ CD62L^-^ OT-1 T cells (Fig 3D). These results indicated that tumor-infiltrating CD62L^+^ T cells also had a high potential to expand.

### Bcl6 expression in CD62L^+^ tumor-infiltrating T cells is critical for their ability to expand

**Fig 4:**
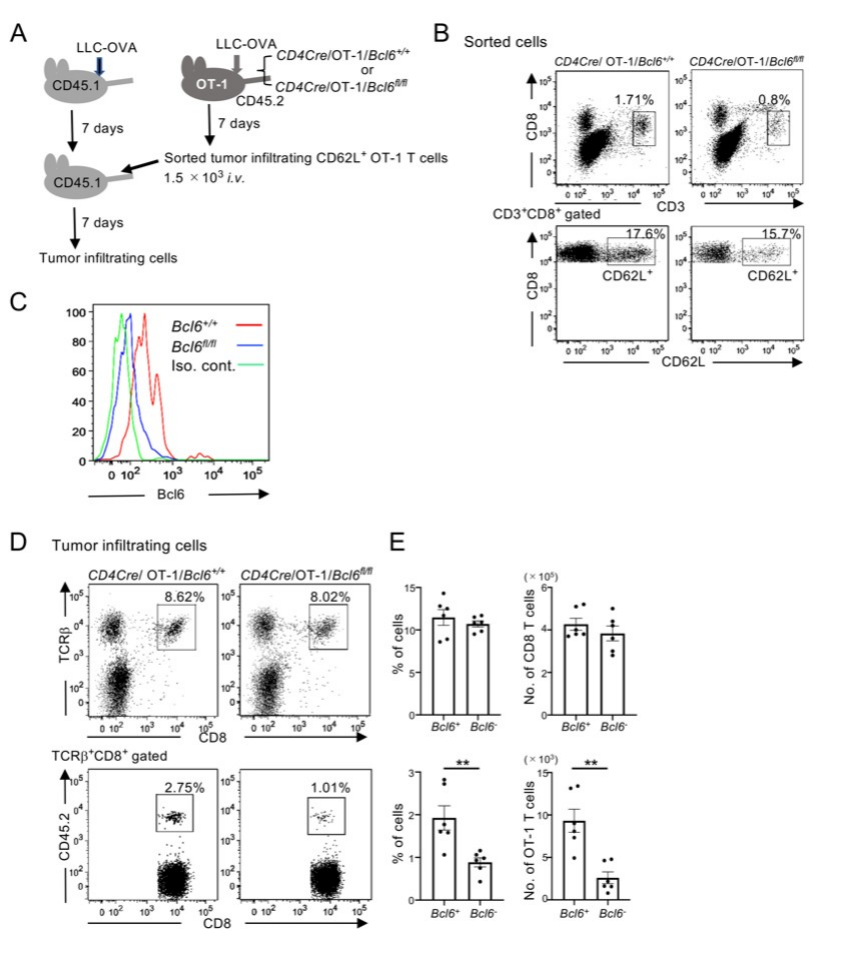
Bcl6 expression in CD62L^+^ tumor-infiltrating CD8 T cells is required for their high proliferative potential. (A) Experimental design. *CD4Cre*/OT-1 mice with either wild-type or floxed Bcl6 alleles (CD45.2) were transplanted with LLC-OVA, and tumor-infiltrating CD62L^+^ OT-1 T cells were sorted on day 7. Cells were transferred *i*.*v*. into LLC-OVA–transplanted C57BL/6 mice (CD45.1), and tumor-infiltrating cells were analyzed on day 7. (B) Representative flow cytometric profiles of sorted CD62L^+^OT-1 T cells of three independent experiments are shown. (C) Bcl6 expression of sorted CD62L^+^ tumor-infiltrating cells in Bcl6 wild-type (*Bcl6*^*+/+*^) and Bcl6-floxed (*Bcl6*^*fl/fl*^) OT-1 T cells in a representative experiment of two independent experiments is shown. (D) (E) Representative flow cytometric profiles of tumor-infiltrating cells 7 days after CD62L^+^OT-1 T cell transfer. Three independent experiments were performed by using two recipient mice per group in each experiment. Frequencies and cell numbers of tumor-infiltrating TCRβ^+^CD8 T cells (top) and CD45.2^+^ (transferred) cells among TCRβ^+^CD8 T cells (bottom) from the pooled data are shown. Mean ± SEM; n = 6; **P<0.01.

Because tumor-infiltrating CD62L^+^ cells expressed Bcl6, we assessed the role of Bcl6 using *Cd4-Cre*/*Bcl6*-*floxed* mice. To this end, we transplanted LLC-OVA tumor cells into Bcl6-deficient OT-1 or wild-type OT-1 (CD45.2) mice, and then 7 days later sorted tumor-infiltrating CD62L^+^ OT-1 T cells (Fig 4A and 4B). Although *Bcl6*-*floxed* OT-1 mice had fewer tumor infiltrating cells, CD62L^+^ cells were found in both mice with similar frequencies (Fig 4B). Sorted CD62L^+^ OT-1 T cells from *Bcl6*^*+/+*^ and *Bcl6*^*fl/fl*^ mice (Fig 4C) were transferred into LLC-OVA–transplanted CD45.1 wild-type mice, and tumor-infiltrating cells were analyzed 7 days later. In mice that received wild-type OT-1 T cells, around 3% of tumor-infiltrating CD8 T cells were transferred cells, whereas Bcl6-deficient OT-1 T cells could reconstitute around 1% of tumor infiltrating CD8 T cells (Fig 4D). Transferring CD62L^+^ tumor-infiltrating cells with Bcl6 deficiency resulted in a significant reduction in both frequencies and cell numbers in the recipient mice (Fig 4D and 4E). These results demonstrated that Bcl6 expression in CD62L^+^ tumor-infiltrating cells is relevant to their ability to expand.

### CD62L^+^BCL6^+^ CD8 T cells become effector T cells to control tumor growth

**Fig 5:**
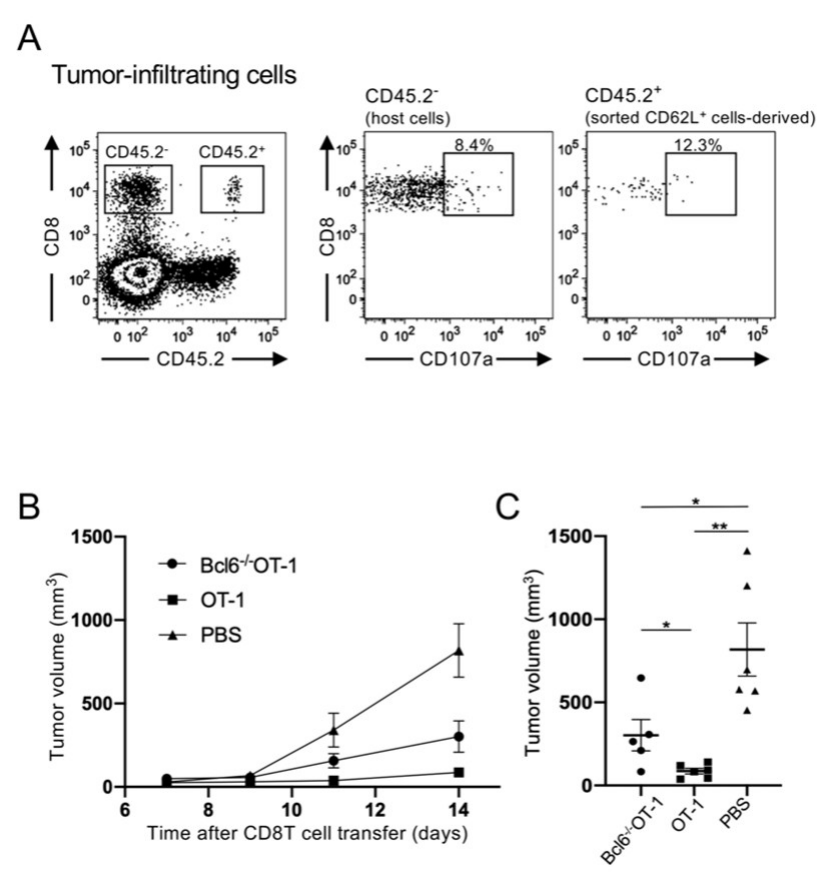
CD62L^+^Bcl6^+^ tumor-infiltrating cells exhibit cytotoxicity. (A) Tumor-infiltrating CD62L^+^ and CD62L^-^ CD8 T cells were sorted from OT-1 mice (CD45.2) that had been transplanted with B16-OVA one week in advance. Sorted cells were then transferred into B16-OVA-transplanted wild-type mice (CD45.1). CD107a expression in tumor-infiltrating CD8 T cells derived from transferred CD62L^+^ cells (CD45.2^+^) and host T cells (CD45.2^-^) were analyzed. One representative analysis of two independent experiments is shown. (B) C57BL/6 mice (6 week-old-female) were subcutaneously transplanted with 1×10^5^ B16-OVA cells. One day later, 1 ×10^6^ OT-1 T cells either from Bcl6^+/+^ or Bcl6^-/-^ mice that had been activated with 100 pM OVA peptides for 48 h were transferred *i*.*v*. and tumor volumes were analyzed on the day indicated after the tumor transplantation. Representative analysis of two independent experiments is shown. PBS was injected as a control. Mean ± SEM; n = 6 (C) Tumor volumes on day 14 in the individual mice are shown. Error bars are Mean ± SEM; n = 6; *P<0.05; **P<0.01.

To assess the role of CD62L^+^Bcl6^+^ tumor infiltrating CD8 T cells, effector functions to control tumor growth were analyzed. First, we tested whether CD62L^+^Bcl6^+^ CD8 T cells become cytotoxic. Tumor infiltrating CD62L^+^ and CD62L^-^ CD8 T cells were sorted from OT-1 (CD45.2) mice and transferred into wild-type (CD45.1) mice, which had been transplanted with B16-OVA. Six days later, transferred OT-1 T cells (CD45.2^+^) that had been infiltrated in the tumor were analyzed. OT-1 T cells sorted as CD62L^+^ T cells were found to be cytotoxic within the recipient tumors. Around 12% of those T cells expressed CD107a, the frequency was similar to that in host CD8 T cells (Fig 5A). In contrast, those sorted as CD62L^-^ T cells were hardly detected within the tumor as shown in Fig 3D. These results suggested that CD62L^+^Bcl6^+^ tumor-infiltrating CD8 T cells had more potential to kill tumor cells than CD62L^-^Bcl6^-^ cells when they were transferred. To see whether CD62L^+^ T cells contribute to control tumor growth, C57BL/6 mice were transplanted with B16-OVA. OT-1 T cells from Bcl6^+/+^ and Bcl6^-/-^ mice were activated *in vitro* by using the OVA peptide and transferred into tumor-transplanted mice. In mice that received Bcl6^+/+^OT-1 T cells, tumor growth was well controlled, whereas larger tumors were detected in those received Bcl6^-/-^OT-1 T cells (Fig 5B and 5C). Since Bcl6 was an essential factor for CD62L^+^ T cell expansion, it was suggested that CD62L^+^Bcl6^+^ T cells within the tumor played a relevant role to control tumor growth.

## Discussion

Cytotoxic CD8 T cells targeting tumor cells play essential roles in tumor immunity. In chronic viral infection and tumor immunity, Tcf1^+^ memory-like CD8 T cells or CD62L^+^ CD8 T cells act as self-renewing stem-like cells that respond to anti–PD-1 checkpoint blockade [8, 9]. Previously, however, it was not known how these memory-like cells were generated. In this study, we demonstrated that CD62L^int^CD44^high^ cells, which appeared 24 h after antigen priming, gave rise to both tumor-infiltrating CD62L^-^ effector and CD62L^+^ memory-like cells. Because these CD62L^int^CD44^high^ cells, as well as CD62L^low^CD44^high^ and CD62L^high^CD44^high^ cells, were already circulating 72 h after priming, it is very likely that CD62L^int^CD44^high^ cells directly infiltrated into the tumor. CD62L^int^CD44^high^ cells in tumor-draining lymph nodes expressed Ki67 and kept proliferating for at least 7 days after antigen priming. These cells could serve as a source of tumor-infiltrating CD62L^+^Bcl6^+^ cells for a long period of time, even after the effector cells became exhausted. It has been shown that during chronic viral infection, antigen-presenting cells preferentially induce less activated Tcf1^+^ CD8 T cells [7]. It remains to be determined whether additional signals are required to progress from CD62L^int^CD44^high^ to CD62L^low^CD44^high^ effector cells.

In tumor-draining lymph nodes, proliferation of antigen-primed CD8 T cells was initiated in cells in fraction III, which express intermediate levels of CD62L. Our results were in agreement with cell-cycle tracking of antigen-primed CD8 T cells, which proliferate rapidly in the CD62L^intermediate^ state, and then split into CD62L^-^ effector and CD62L^high^ central memory T cells [22]. Differentiation toward effector and memory T cells can be driven by asymmetric partition of fate-determining proteins in the first division of antigen-primed CD8 T cells, resulting in differential activation of mTOR and leading ultimately to effector versus memory T cell differentiation [23]. Heterogeneity of gene expression after the first division has been also reported [2].

Bcl6 expression in naïve CD8 T cells is reported to be downregulated after antigen stimulation [4]. It is possible that tumor-infiltrating Bcl6^+^CD62L^+^ cells re-expressed Bcl6 while the expression of Bcl6 in CD62L^-^ effector cells remained repressed by continuous stimulation. Tumor-infiltrating CD62L^+^Bcl6^+^ cells possessed a high potential to proliferate. The lack of Bcl6 in CD62L^+^ cells decreased their proliferative potential, which could be due to the reduced function of Tcf1. Bcl6 promotes the expression of Tcf1 [14], which is required for memory T cell generation and expansion [24]. Because terminally differentiated effector cells lose the proliferative potential, CD62L^+^Bcl6^+^ cells within the tumor must play a pivotal role in replenishing anti-tumor CD8 T cells. Thus, promotion of continuous proliferation and self-renewal of CD62L^int^CD44^high^ cells in secondary lymphoid tissues could help supply CD62L^+^Bcl6^+^ cells within the tumor, augmenting anti-tumor immunity.

## Supporting information

S1 Fig Part of Tcf1^+^ cells in tumor-infiltrating CD8 T cells express Bcl6. Tumor infiltrating CD8 T cells in B6-OVA transplanted tumors were analyzed. Representative flow cytometry plots and histograms of three independent experiments are shown.
(TIFF)

S2 Fig Tumor-infiltrating polyclonal CD62L^+^ CD8 T cells express CXCR3 and lack PD-1. C57/BL6 mice were transplanted with LLC-OVA and tumor infiltrating TCRβ^+^CD8 T cells were analyzed. (A) One representative flow cytometry plot of three independent experiments is shown. (B) PD-1 expression in CD62L^+^ and CD62L^-^ tumor-infiltrating TCRβ^+^CD8 T cells was analyzed three weeks after tumor transplantation. Representative data of three independent experiments are shown.
(TIFF)

S3 Fig Detection of antigen-primed OT-1 T cells in the spleen. Spleen cells in LLC-OVA transplanted C57BL/6 (CD45.2) mice, shown in Fig 2A, were analyzed 24h, 48h and 72h after naïve OT-1 T cell transfer. CD8^+^CD45.1^+^ cells were gated as in Fig 2B based on the expression of CD44 and CD62L (Fr. III, IV and V).
(TIFF)

S4 Fig Expression levels of Ki67 in CD44^high^ fractions on day 7. OT-1 mice were transplanted with LLC-OVA. Tumor-draining lymph node cells, gated on CD3^+^CD8^+^, were sorted into three fractions; CD62L^int^CD44^high^ (III), CD62L^low^CD44^high^ (IV) and CD62L^high^CD44^high^ (V). Sorted cells were fixed and stained with anti-Ki67. One representative analysis of three independent experiments is shown.
(TIFF)
