# Supplementary figures and images for: Tumor-infiltrating CD62L+PD-1-CD8 T cells retain proliferative potential via Bcl6 expression and replenish effector T cells within the tumor
Source: PLoS One. 2020 Aug 26;15(8):e0237646. doi: 10.1371/journal.pone.0237646 (PMC7449457; doi:10.1371/journal.pone.0237646)

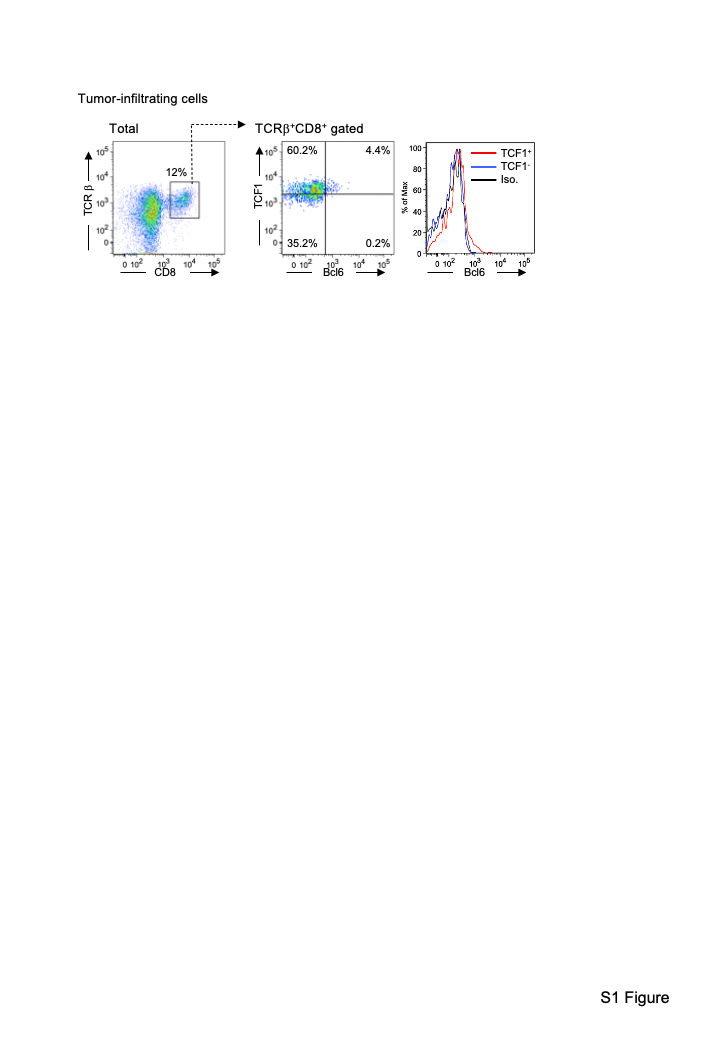

Supplement: S1 Fig — Tumor infiltrating CD8 T cells in B6-OVA transplanted tumors were analyzed. Representative flow cytometry plots and histograms of three independent experiments are shown. (TIFF) [file pone.0237646.s001.tiff]

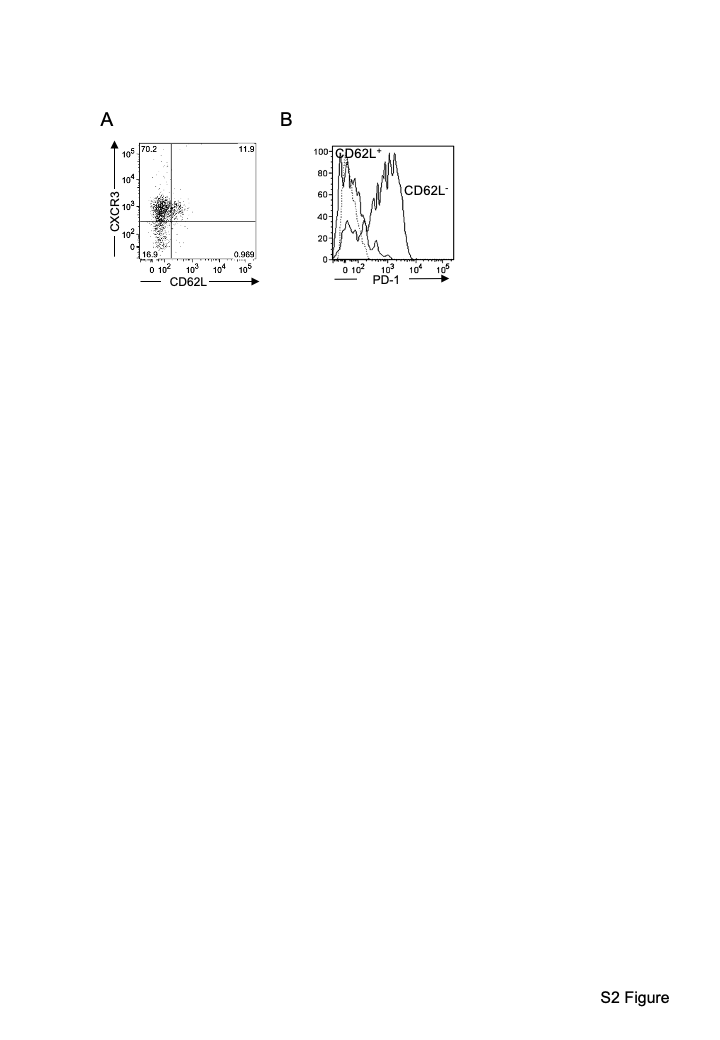

Supplement: S2 Fig — C57/BL6 mice were transplanted with LLC-OVA and tumor infiltrating TCRβ+CD8 T cells were analyzed. (A) One representative flow cytometry plot of three independent experiments is shown. (B) PD-1 expression in CD62L+ and CD62L- tumor-infiltrating TCRβ+CD8 T cells was analyzed three weeks after tumor transplantation. Representative data of three independent experiments are shown. (TIFF) [file pone.0237646.s002.tiff]

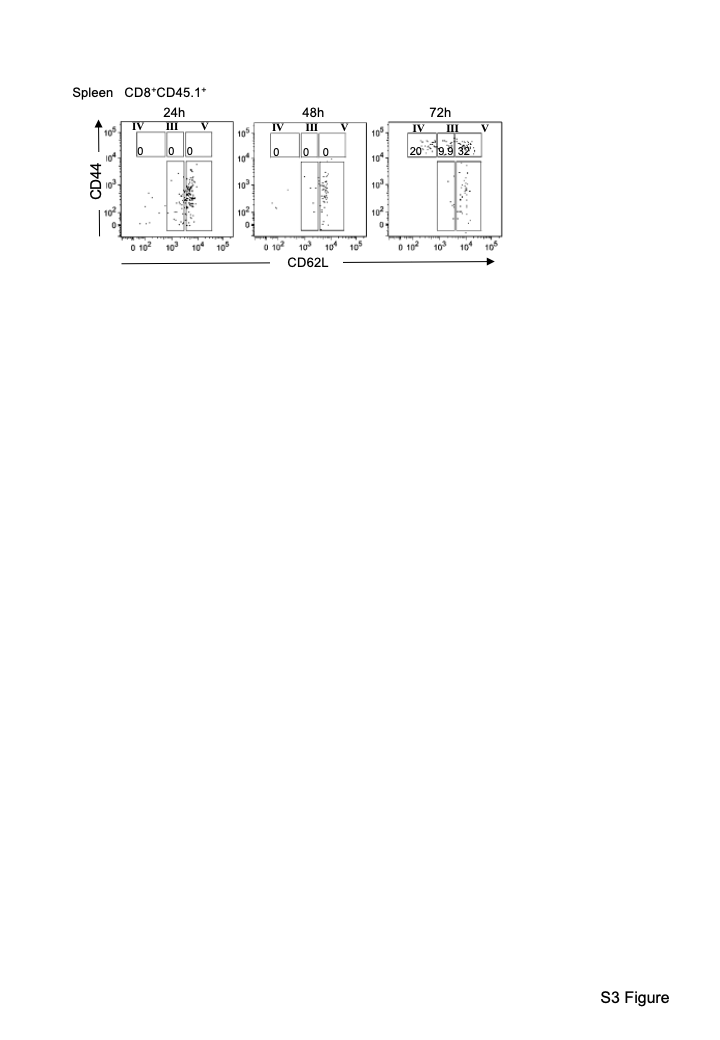

Supplement: S3 Fig — Spleen cells in LLC-OVA transplanted C57BL/6 (CD45.2) mice, shown in Fig 2A, were analyzed 24h, 48h and 72h after naïve OT-1 T cell transfer. CD8+CD45.1+ cells were gated as in Fig 2B based on the expression of CD44 and CD62L (Fr. III, IV and V). (TIFF) [file pone.0237646.s003.tiff]

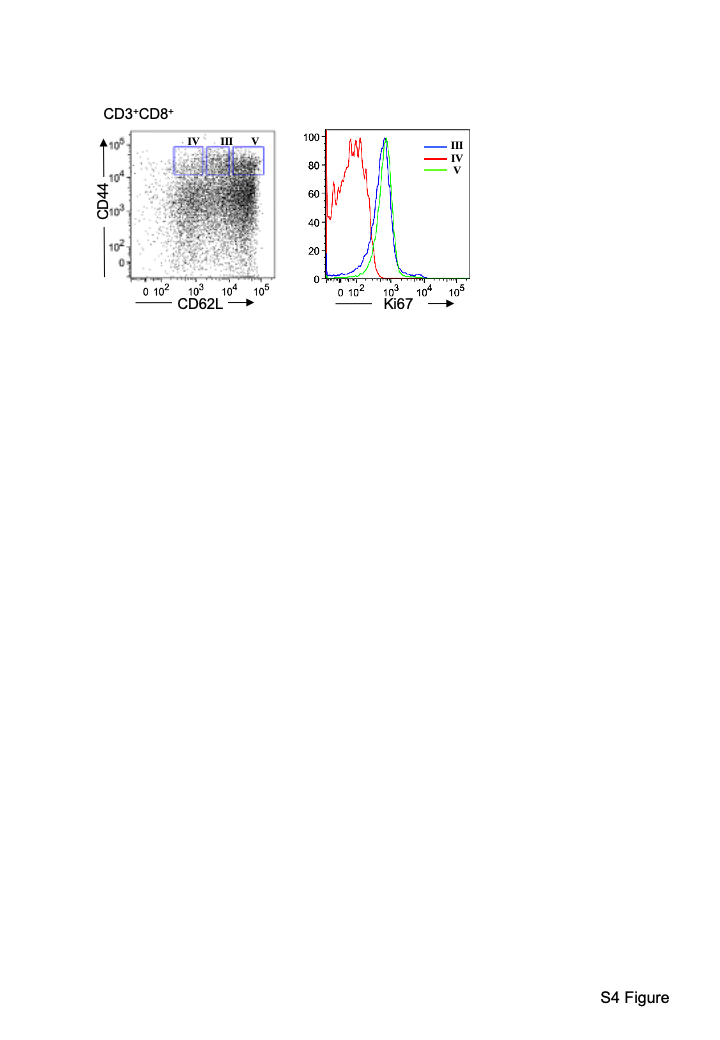

Supplement: S4 Fig — OT-1 mice were transplanted with LLC-OVA. Tumor-draining lymph node cells, gated on CD3+CD8+, were sorted into three fractions; CD62LintCD44high (III), CD62LlowCD44high (IV) and CD62LhighCD44high (V). Sorted cells were fixed and stained with anti-Ki67. One representative analysis of three independent experiments is shown. (TIFF) [file pone.0237646.s004.tiff]
